# Optimization of BRET saturation assays for robust and sensitive cytosolic protein–protein interaction studies

**DOI:** 10.1038/s41598-022-12851-9

**Published:** 2022-06-15

**Authors:** Benoit Besson, Hyeju Eun, Seonhee Kim, Marc P. Windisch, Herve Bourhy, Regis Grailhe

**Affiliations:** 1 Technology Development Platform, Institut Pasteur Korea, 16, Daewangpangyo-ro 712 beon-gil, Bundang-gu, Seongnam-si, Gyeonggi-do 463-400 Republic of Korea; 2 Institut Pasteur, Unité Dynamique des Lyssavirus et Adaptation à l’Hôte, 28 rue du docteur Roux, 75015 Paris, France; 3 Université Paris Diderot, Sorbonne Paris Cité, Cellule Pasteur, rue du Docteur Roux, 75015 Paris, France; 4 Applied Molecular Virology, Institut Pasteur Korea, 16, Daewangpangyo-ro 712 beon-gil, Bundang-gu, Seongnam-si, Gyeonggi-do 463-400 Republic of Korea

**Keywords:** Cell signalling, Virus-host interactions, Proteomics, High-throughput screening, Imaging, Microscopy, Proteomic analysis, Fluorescent proteins

## Abstract

Bioluminescence resonance energy transfer (BRET) saturation is a method of studying protein–protein interaction (PPI) upon quantification of the dependence of the BRET signal on the acceptor/donor (A:D) expression ratio. In this study, using the very bright Nluc/YFP BRET pair acquired respectively with microplate reader and automated confocal microscopy, we significantly improved BRET saturation assay by extending A:D expression detection range and normalizing A:D expression with a new BRET-free probe. We next found that upon using variable instead of fixed amount of donor molecules co-expressed with increasing acceptor concentrations, BRET saturation assay robustness can be further improved when studying cytosolic protein, although the relative amounts of dimers (BRETmax) and the relative dimer affinity (BRET50) remain similar. Altogether, we show that our method can be applied to many PPI networks, involving the NF-κB pathway, high-affinity nanobody, rabies virus-host interactions, mTOR complex and JAK/STAT signaling. Altogether our approach paves the way for robust PPI validation and characterization in living cells.

## Introduction

The increasing complexity and amounts of protein–protein interactions (PPI) involved in cellular responses foster the constant development of novel technologies to monitor the strength and dynamics of these interactions. When studying native PPIs occurring in living cells, fluorescence and bioluminescence resonance energy transfer (FRET/BRET) techniques are particularly powerful. Indeed, resonance energy transfer quantification relies on the measurement of increased acceptor emission intensity occurring upon proximity with acceptor molecules (Supplementary Fig. S1) and varying as the inverse sixth power of the distance between A:D^1^. Noticeably, BRET replaces the fluorescent donor with a bioluminescent protein, which does not depend on excitation illumination, circumventing per se major FRET drawbacks^2^.

BRET saturation assays were developed (sometimes referred as qBRET^3^) to monitor, quantify, and compare PPIs pairs. Using this method, the BRET and ratiometric expression of bioluminescent-tagged (bait) and fluorescent-tagged (prey) proteins were quantified in living cells. Whether using a stable expression of donor associated with variable concentration of acceptor^4,5^ or varying the expression of both donor and acceptor^3,6,7^, BRET saturation assays are plotted as a function of A:D expression ratio^3,6,7^ and as such, require both acceptor and donor expression to be accurately monitored. However, discrepancies in the detection methods that favor bioluminescence probes compared to fluorescence probes can be found. As a result, the detection of the fluorescent acceptor probe constitutes a bottleneck in BRET saturation assays, causing narrow A:D detection ranges.

In this study, several key improvements are proposed to quantify A:D protein pairs accurately and allow broader use of BRET saturation assays for PPI research. Taking advantage of donor Nluc brightness^8,9^ acquired with a luminescence plate reader and the increased sensitivity for acceptor YFP using automated confocal microscopy as an alternative detection method, we achieved high donor and acceptor detection sensitivity as well as robust BRET signal across a broad range of A:D expression ratios.

Focusing on cytosolic proteins that exhibit heteromeric PPIs and negligible non-specific BRET, we compared BRET saturation assay upon stable expression of donor associated with variable expression of acceptor or upon varying the expression of both donor and acceptor. We found that variable expression of both donor and acceptor BRET saturation assays provides a better range for acceptor detection while providing similar BRETmax and BRET50 compared to the fixed donor expression method. We next applied our BRET saturation method to various models as follow: the NF-κB pathway featuring homo-and hetero-dimeric interactions and mutants of rabies virus matrix (M) protein showing different affinities with a quaternary protein complex part of the NF-κB pathway^10^, well-characterize nanobody-YFP and SOD1 dimer interactions, interactions regulated by chemicals within the mTOR complex, and the JAK/STAT pathway and its IFN-dependent signal transduction leading to the formation of ISGF3.

## Results and discussion

### Optimization of BRET saturation assays

**Figure 1:**
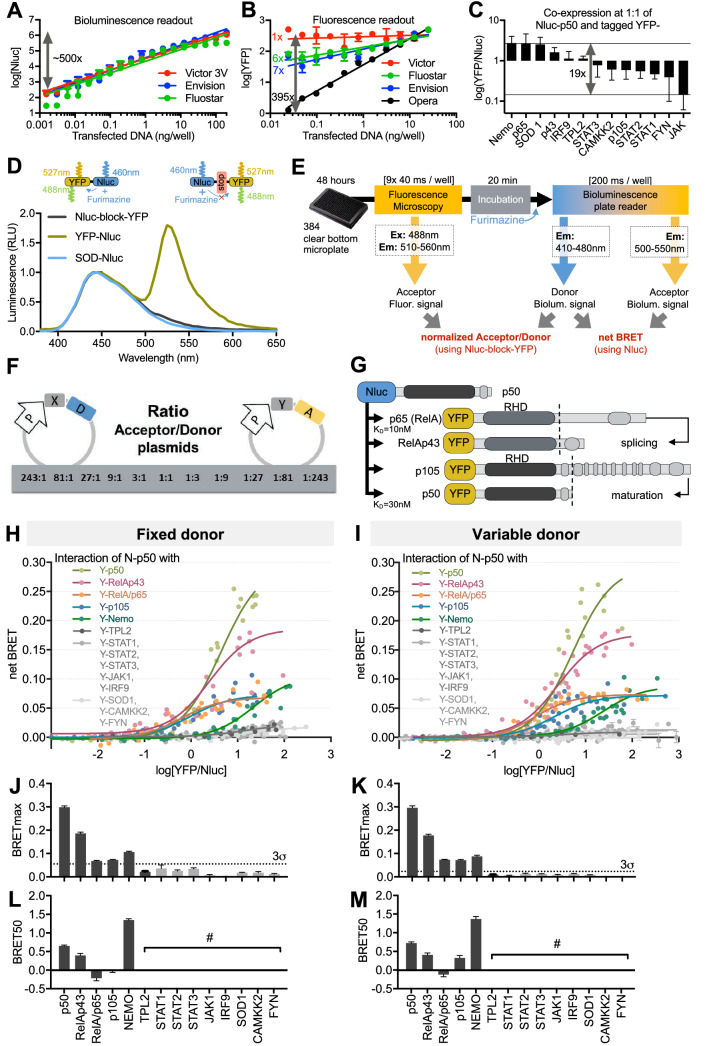
A sensitive method for PPI interaction studies based on BRET saturation assays. (**A**,**B**) HEK-293T cells were transfected with 0.025 to 25 ng of YFP-Nluc plasmid per well. Nluc bioluminescence was quantified using various microplate readers (**A**), and YFP fluorescence was quantified using microplate readers or an automated confocal microscope (**B**). (**C**) YFP and Nluc signals were sequentially quantified for 13 sets of plasmids transfected in HEK-293T cells at 1:1 A:D ratios. (**D**) Bioluminescence spectra properties of reference proteins. Results are the average of three independent experiments. (**E**) Sequential measurement of the fluorescence of the acceptor (with an automated microscope) and the bioluminescence emitted from both A:D upon BRET (with a plate reader) in order to calculate the net BRET and the A:D expression ratio using the BRET-free Nluc-block-YFP control for BRET saturation assays. (**F**) Broad range transfection method for BRET saturation assay over 11 ratios (243:1 to 1:243, see Supplementary Fig. S2). (**G**) Diagram of NF-κB protein domains and sequence homologies. (**H**,**I**) BRET saturation assay in HEK-293T cells transfected with the donor (Nluc-p50) and acceptor (YFP-tagged) plasmids using fixed (**H**) or variable (**I**) donor. The net BRET was plotted with the YFP/Nluc expression ratios and fitted using a non-linear regression curve to determine both BRETmax and BRET50. The results represent four independent biological replicates, each involving three technical replicates. BRETmax values (**J**,**K**) were used to define interacting, non-interactive pairs (grey labeled) and extrapolate a 3 σ-threshold [(**J**) 0.055 and (**K**) 0.023]. BRET50 values were reported only for significant interacting pairs (**L**,**M**). # not displayed.

BRET saturation assays can potentially be subjected to experimental or analytical pitfalls as the A:D expression ratio needs to be accurately monitored and analyzed, as previously highlighted^3,6,11^. For this reason, the sensitivity of fluorescent and bioluminescent plate readers commonly used^5,7,11^ were assessed using a fluorescent and bioluminescent reference probe ‘YFP-Nluc’ (Fig. 1D). For all tested plate readers, we found a linear relationship between the transfected material and the bioluminescence signal of Nluc with a dynamic range of ~ 500 fold (Fig. 1A) while the detection sensitivity for YFP was considerably reduced (< 10-fold, Fig. 1B). This lack of sensitivity presents severe limitations when applying the BRET saturation method as it does not provide enough resolution. In comparison, automated confocal microscopy extended the detection sensitivity of YFP expressing plasmid down to 0.025 ng of DNA/well, increasing the dynamic range to 395-fold (Fig. 1B). Meanwhile, the YFP-Nluc BRET signal remained constant, and the A:D signal relationship remained linear across a broad range of expression (Supplementary Fig. S1).

In light of the protein expression variability for any given plasmid transfected (Fig. 1C), it is key to precisely determine the relative expression of donor and acceptor protein in the cellular model of interest. To circumvent the attenuation of the 460 nm Nluc signal from the Nluc-YFP probe due to energy transfer (Fig. 1D) while maintaining a 1:1 A:D ratio reference ratio, we introduced a large rigid peptide between Nluc and YFP, generating a novel tandem protein named Nluc-block-YFP (Fig. 1D). As expected, our new Nluc-block-YFP reference probe emits a bioluminescence spectrum comparable to Nluc alone and is free of any BRET signal, therefore providing a proper equimolar donor and acceptor signals across a broad range of expression (Supplementary Fig. S1).

With extended detection and normalization of the A:D ratios, we propose a novel protocol for BRET saturation assay amenable to evaluate PPI of numerous protein pairs tested in a high-throughput screening setup (Fig. 1E). Experiments are performed in flat bottom μClear plates permitting the quantification of the fluorescence signal of the acceptor YFP by automated microscopy. Cells are then incubated before adding furimazine and measuring the bioluminescence signal of the donor Nluc and acceptor YFP with a plate reader. Both fluorescent and bioluminescent signals are normalized separately using the Nluc-block-YFP reference probe expressing acceptor and donor at a fixed ratio of 1:1 in order to determine the ratio of Nluc- and YFP-tagged proteins of interest. Nluc control is then used to correct for the bleed-through signal of Nluc and calculate the net BRET signal. Exploiting Nluc brightness for donor emission and microscopy sensitivity for acceptor detection, we next propose to broaden the A:D expression plasmids ratio from 243:1 to 1:243 in 3 step dilutions (Fig. 1F, Supplementary Fig. S2). In comparison to previous reports applying BRET saturation assays with A:D expression ranging from 1:2 to 66:1^6^ or 1:1 to 250:1^11^, such extended range allows performing BRET saturation assay on protein pair regardless of their relative expression level discrepancy as shown in Fig. 1C.

As a result, upon combining confocal microscopy for detection of acceptor YFP and plate reader for detection of extremely bright BRET donor Nluc, we extended the detection range of A:D pairs and enabled to use a broader range of transfected A:D expression plasmid ratios. Altogether, we suggest applying our improved methodology based on microscopy and Nluc-block-YFP for all BRET saturation assays used for both cytosolic and membrane proteins.

### Comparison between fix donor versus variable donor BRET saturation assays with NF-κB protein network pairs

Fixed^4,5^ and variable donor^3,6,7,12^ BRET saturation assays have been well described for membrane proteins yet were never compared directly for soluble proteins. Taking advantage of our optimization for BRET saturation assays, we compared both methods, focusing on cytosolic proteins for which the requirements for donor ‘constant density’ do not apply^13^ therefore limiting the use of variable donor BRET saturation assay for membrane proteins^3,6,7,12^. Within the NF-κB pathway, we selected p65 (RelA), RelAp43, p50 and p105^10,14–16^ known to interact with p50 (Fig. 1G), and NF-κB regulatory proteins NEMO (IKKγ) and TPL2 for which no direct interaction with p50 was reported^14,17^. Outside of the NF-κB pathway, we chose proteins belonging to the JAK/STAT signaling pathway (STAT1, 2, and 3, JAK1 and IRF9) and random selections from our collection (SOD1, CAMKK2, and FYN), which have not been reported to interact with p50. Upon computing non-linear regression curves (Fig. 1H,I), two key parameters can be quantified (Supplementary Fig. S2): (i) the BRETmax, which represents the maximal BRET signal reached at donor saturation (Fig. 1J,K), and (ii) the BRET50, which corresponds to the [YFP]/[Nluc] ratio giving 50% of BRETmax (Fig. 1L,M). It should be noted that if BRET50 correlates with protein–protein affinity, unlike a K_D_, it is a unitless, and we will refer to its properties as “relative affinity”. Further, while a K_D_ is determined in a controlled environment in vitro, BRET50 is measured in a heterogeneous and protein dense cytosol environment where specific physicochemical conditions may affect PPIs.

Our results show that Nluc-p50 co-expressed with YFP-p50, -p65, -RelAp43, -p105 and -NEMO produced robust hyperbolic BRET saturation curves when compared to non-interacting protein pairs (grey). Amongst NFκB proteins, RelAp43 is a splicing variant of p65 and p50 is the matured form of p105 (Fig. 1G), therefore RelAp43/p65 and p50/p105 share identical Rel homology domains (RHD) responsible for their homo/hetero-dimerization. Along with identical N-ter sequences, each pair is tagged with the same acceptor in N-ter position and therefore only differ in their C-ter sequence. BRETmax comparison suggests that p50-p50 and p50-p105, as well as p50-RelAp43 and p50-p65, do not share the same interacting properties. Likely, the proximity or conformation differences of the proteins lead to an alteration in the A:D distance and/or their orientation. BRET50 comparison suggests a higher relative affinity of p65- and p105-p50 versus RelAp43- and p50-p50, which correlates with affinities measured in vitro (p50-p65 K_D_ = 10 nM, p50-p50 K_D_ = 30 nM)^18^. Such results provide new insights into the NF-κB pathway dynamics and especially on the role of RelAp43 as a potent p65 competitor^10,15,16^. Additionally, the p50-NEMO BRET saturation curve suggests that both proteins are interacting, which has never been reported and remains to be confirmed. However, while NF-κB dimers consist of known direct interactions, p50-NEMO high BRET50 suggests a low-affinity interaction, which may occur within a broader molecular complex. Inversely, we did not observe any significant interaction between p50 and TPL2, although molecular complexes have been previously reported^19^. These data highlight both the specificity and the sensitivity of the method. Finally, BRETmax values obtained from the non-interacting protein pairs can be used as negative controls to set a 3σ threshold in BRET saturation assays to exclude non-specific interactions (Fig. 1J,K).

Interestingly, both methods (fix or variable donor) provide similar BRET saturation profiles (Fig. 1H,I). However, we found that BRET saturation assay based on the transfection of a fixed donor was prone to failure when transfection efficiency was reduced. To compare the robustness of both methods undertaking transfection efficiency fluctuation, we performed a series of BRET ratio assays with a variable total amount of plasmid transfected per well using the Nluc-p50/YFP-tagged p50 pair as a model (Supplementary Fig. S3). BRET saturation curve fitting was possible only when using 50 ng total plasmids for the fixed donor BRET saturation. However, we found that the variable donor method reached a proper signal saturation reliably, even when transfecting 16 times fewer plasmids. It should be noted that BRETmax and BRET50 obtained with the latter are subject to minor variations at lower concentrations of plasmid transfected, possibly due to possible competition with endogenous proteins.

Such robustness differences between the BRET saturation ratio using fixed and variable acceptor concentration could be explained as the range of detection of the acceptor was significantly lower with fixed donor (5–8 ratios) compared to variable donor (10–11 ratios, Supplementary Fig. S4). Furthermore, although the donor plasmid was transfected at a fixed concentration, the Nluc bioluminescent signal significantly and systematically decreases in particular for high A:D plasmid ratios. Such result defeats one of the main interests of the fix donor method, developed to determine the homomeric stoichiometry of proteins based on a single variable^6^. We found that the decrease of expression of the Nluc at the highest concentration of acceptor was not related to energy transfer events as the same phenomenon was found with non-interacting BRET pairs (Supplementary Fig. S5). We believe that the decrease of Nluc-tagged protein was rather due to reduced co-transfection efficiency or limited RNA polymerase (trans or competition) between promoter of the A:D plasmids. Altogether, our data demonstrate that using a variable expression of both donor and acceptor, BRET saturation assays provide a better range for acceptor detection and improves the robustness of the assay, either to study known or to screen for novel partners.

### BRETmax and BRET50 describe PPIs rationally

**Figure 2:**
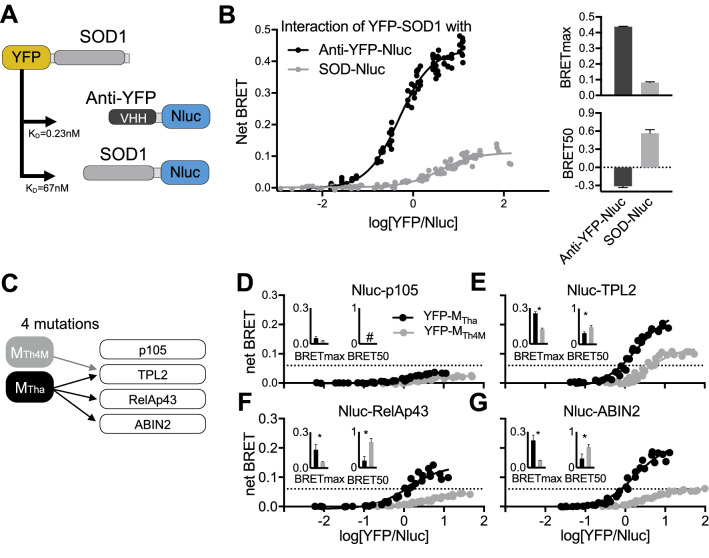
Variable donor BRET saturation assay applied to study protein complex with distinctive affinities. (**A**) Known interaction parameters of YFP and anti-YFP nanobody or SOD1 dimers. (**B**) Variable donor BRET saturation assay of donor SOD-Nluc or anti-YFP-Nluc with acceptor YFP-SOD1. The net BRET was plotted with the YFP/Nluc expression ratios to determine both BRETmax and BRET50. (**C**) Rabies virus M_Tha_ and M_Th4M_ mutant proteins interact differentially with host proteins p105, TPL2, RelAp43, and ABIN2. (**D**–**G**) Variable donor BRET saturation assay of donor Nluc-p105 (**D**), -TPL2 (**E**), -RelAp43 (**F**) or -ABIN2 (**G**) and acceptor (YFP-M_Tha_ or M_Th4M_) as in Fig. 1 and both BRETmax and BRET50 were plotted. A 3σ-threshold (0.06) was defined, based on the M-p105 interactions considered as negative controls. # not displayed. Unpaired, parametric two-tailed *t* test were performed with GraphPad (Prism), *p < 0.05.

To validate the sensitivity of our approach, we next performed a series of BRET saturation assays using different PPIs with distinctive affinities using the microscopy-based variable donor ratio method exclusively. A single variable domain on a heavy chain (VHH) antibody or nanobody with a high affinity to YFP and GFP proteins (K_D_ = 0.23 nM)^20^ was fused to a Nluc (anti-YFP-Nluc). SOD1, which can efficiently form homo-dimers (K_D_ = 67 nM), was linked to Nluc and YFP (Fig. 2A). Donor anti-YFP-Nluc and SOD1-Nluc interaction with acceptor YFP-SOD1 were assessed in a BRET saturation assay (Fig. 2B). BRETmax for the anti-YFP-Nluc/SOD1-YFP BRET pair was found superior (0.43) as compared to the SOD1 homodimers (0.11), likely due to a closer proximity of the bioluminescence and fluorescence proteins. The BRET saturation curve shifted to the left, reflecting a significantly higher relative affinity of the anti-YFP nanobody (BRET50 = − 0.3) compared to SOD1 homodimers (BRET50 = 0.6). The association of all dimers at a A:D ratio of 1:1, representing the minimum BRET50 theoretically possible, suggests that BRET saturation assays are limited in the resolution of high affinity interactions, where subtle changes in relative affinity will not be measurable. This also highlight the necessity to start dilutions at low A:D ratios (down to 1:243) when studying high affinity interactions compared to previous methods starting at 1:1 or 1:2^6,11^.

To further illustrate the sensitivity of our method to study the relative affinity between protein using BRET50 quantification, we selected the matrix (M) protein of rabies virus Thailand (Tha) and mutant (Th4M)^21^. Compared to M_Tha_, M_Th4M_ encodes four substitutions which do not affect viral replication^16^ and suggests that the protein structure is not significantly affected, making it a valid model for BRET analysis. Interestingly, M_Th4M_ mutations impair its capacity to interact with the RelAp43-p105-ABIN2-TPL2-p105 complex, related to NF-κB signaling (Fig. 2C)^10^. Indeed, if M_Tha_ and M_Th4M_ interact with TPL2 while only M_Tha_ interacts with RelAp43 and ABIN2, and neither interacts with p105^10^. Here, we confirm that M protein variants do not interact substantively with p105 (Fig. 2D) while both interacted with TPL2 (Fig. 2E) and exclusively M_Tha_ interacted selectively with RelAp43 (Fig. 2F) and ABIN2 (Fig. 2G). For all interacting pairs, a significant increase of the BRETmax and decrease of the BRET50 synonymous of increased relative affinity and complex formation were observed for Tpl2-, RelAp43, and ABIN2-M_Tha_ as compared to M_Th4M_. At a larger scale, our results demonstrate that our method allows a very reliable comparative PPI analysis.

### Monitoring of chemically regulated PPIs with BRETmax and BRET50

**Figure 3:**
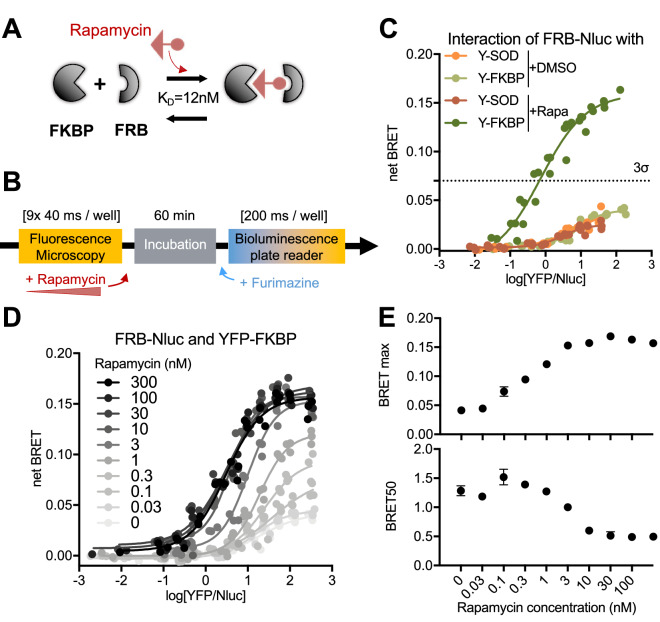
Monitoring the drug-dependent FRB-FKBP interaction with variable donor BRET saturation assays. (**A**) Simplified dynamics of the interaction of FKBP with the FRB domain of mTOR mediated by the rapamycin. (**B**) HEK-293T cells were incubated in presence of rapamycin for 1 h between fluorescence and bioluminescence acquisition. (**C**) Variable donor BRET saturation assay of FRB-Nluc/YFP-FKBP in the presence or absence of rapamycin. A 3 σ-threshold (0.07) was defined based on SOD-FRB interactions considered a negative control. (**D**) Variable donor BRET saturation assay of FRB-Nluc/YFP-FKBP in cells treated with various concentrations of rapamycin for 1 h. (**E**) BRETmax and BRET50 values were plotted according to the different concentrations of rapamycin.

To determine the capability of our improved BRET saturation method to characterize PPI modulators, we selected known PPIs that are regulated by chemicals. First, we studied rapamycin for its ability to bind simultaneously to FKBP and mTOR, a protein kinase involved in the PI3K-Akt pathway (Fig. 3A). Several studies have investigated the affinity between the FKBP-rapamycin-binding (FRB) domain of mTOR and the duplex constituted of rapamycin and FKBP and established that the FKBP-rapamycin-FRB complex has an overall affinity of K_D_ = 12 nM^22^.

Treatment with rapamycin prior to bioluminescence readout (Fig. 3B) led to an increase of the BRETmax in a BRET saturation assay of FRB-Nluc and YFP-FKBP (Fig. 3C). Using incremental concentrations of rapamycin, we measured a progressive increase in BRETmax and decrease in BRET50 (Fig. 3D,E) from 0.46 to 12 nM of rapamycin. Both BRETmax and BRET50 values reached a plateau at 12 nM, which also constitutes the overall K_D_ value of the complex^22^. This result is surprising as only half of the dimers should presumably be formed at the K_D_ concentration, and the BRETmax should have reached only ~ 50% of its peak value. Such variability is likely related to the difference between data obtained in living cells compared to in vitro^22^.

### Time-lapse BRET saturation assay

We next investigated the possibility of performing a time-lapse BRET saturation assay. The YFP-Nluc reference probe BRET signal remained constant over time according to a stepwise dilution of furimazine substrate down to 2000-fold dilution (Supplementary Fig. S6). At lower dilutions, Nluc bioluminescence and BRET signal dropped drastically after 60 min. At such a low concentration of furimazine, repeated measurements over several hours are possible if furimazine is added every hour. On account of a short time-scale experiment (under 1 h), YFP can be measured only once before BRET measurements, and constant A:D ratio values can be set for the whole time-lapse to calculate the BRET50 reliably, granted that the expression of A:D molecules remains constant over the experiment.

**Figure 4:**
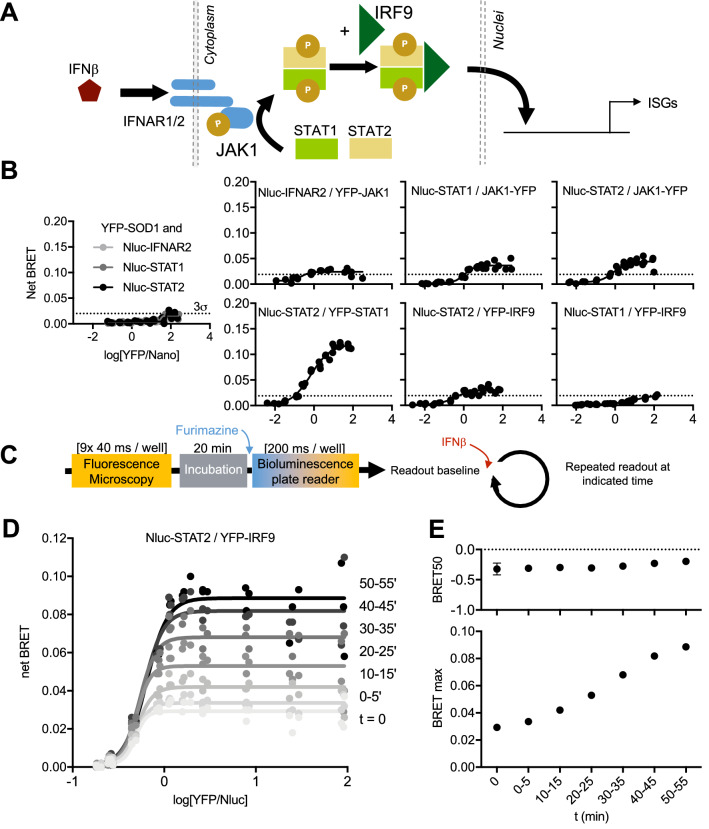
Study of IFN-dependent JAK/STAT pathway and time-lapse monitoring of STAT2/IRF9 interaction. (**A**) JAK/STAT signaling network. (**B**) Variable donor BRET saturation assay of Nluc- and YFP-tagged protein pairs from the JAK/STAT pathway as in Fig. 1. Based on the acceptor protein YFP-SOD1 negative control, a 3σ threshold (0.02) was defined. (**C**) Immediately after the initial bioluminescence acquisition (t = 0), IFNβ was added to the cells at 500 U/mL, and the cells were monitored every 10 min for 1 h. (**D**) Variable donor BRET saturation assay of Nluc-STAT2/YFP-IRF9 after IFNβ stimulation. (**E**) BRET50 and BRETmax were represented according to the time of acquisition.

Next, we selected a set of proteins that belongs to the JAK/STAT signaling pathway (Fig. 4A), known to reorganize upon stimuli^23^. At a resting stage, JAK/STAT proteins are known to co-localize at the membrane vicinity. Upon IFNβ binding to IFNARs, a phosphorylation cascade is induced through JAK1, triggering the phosphorylation and release of STAT1/2 dimers, which binds to IRF9, forming the ISGF3 complex later translocated to the nucleus^23^. A low but significant BRETmax was detected for most pairs tested without IFNβ (Fig. 4B). This could be attributed to an over-expression bias or a basal phosphorylation of a pathway ready to transduce a signal quickly. The only exception was the STAT1/STAT2 dimer (as well as all tested combinations between STAT1, STAT2, and STAT3, data not shown), which showed high BRETmax, suggesting high STAT1/STAT2 dimer formation in the absence of IFNβ as scarcely reported in the literature^24^.

To precisely monitor the signal transduction and the dynamic interaction of STAT2/IRF9, IFNβ was added at t = 0, and the bioluminescent readout was performed every 5 min (Fig. 4C). BRET saturation assay highlights an increase of STAT2-IRF9 interaction in presence of IFNβ (Fig. 4D) through a significant increase of BRETmax over the first hour (Fig. 4E). This represents a sub-population of STAT2 and IRF9 proteins inaccessible for interaction by lack of phosphorylation and released upon IFNβ stimulation to allow the interaction^23^. Notably, both populations prior and after IFNβ treatment showed similar BRET50 (Fig. 4E), thus suggesting similar affinities. Altogether, if used in a properly defined setup, the BRET saturation curve and its derived parameters allow precise monitoring of the kinetic of PPI in stimulated living cells.

## Conclusion

Here, we propose a BRET saturation method that improves the sensitivity and robustness of detection of A:D expression ratios by mean of microscopy and upon usage of a novel BRET free reference probe. Furthermore, we show that it is possible to increase further the BRET saturation assay robustness for cytosolic proteins, which are less subject to non-specific BRET. We found that BRET saturation for non-anchor proteins does not require maintaining a constant donor density, consequently enabling the use of variable expression for both acceptor and donor, further extending the A:D ratio expression. As a result, we demonstrate that such a method applied to soluble protein allows studying the relative affinity ranking between protein pairs, protein mutation effect on PPI, and time dependence over BRET and BRET50 measurements. Altogether, we propose a framework based on microplate format to standardize the BRET saturation method facilitating the PPI quantification in living cells to a broader audience.

## Methods

### Plasmid preparation

The coding region of the human p50, p65, p105, RelAp43, NEMO, TPL2, ABIN2, IFNAR2, JAK1, STAT1, STAT2, IRF9, FYN and CAMKK2 genes were amplified by RT-PCR using RNA preparation from HeLa cells. The FRB domain of mTOR and the YFP-tagged FKBP fusion proteins were obtained from Addgene (plasmid number #31181 and #20175, respectively). The matrix protein-encoding sequences from the wild isolate Thailand of rabies virus (M-Tha) and the matrix mutant on residues 77-100-104-110 (M_Th4M_) were obtained from previous work^15,16^. The pEYFP-C1/N1 plasmids (Clontech) were modified by switching the eYFP by the Nluc (from the pNL1.1, Promega) to obtain new “pNluc-C1/N1” plasmids, adding a tag in N- or C-terminal position. All genes were cloned, leading to N- or C-terminal, Nluc- or YFP-tagged proteins, and the best constructs for known interactions were selected. The anti-YFP-N plasmid was generated upon synthesis of the anti-YFP domain from the Addgene sequence (plasmid #31181) and cloned in pNluc-C1. Two additional plasmids, the chimera YFP-Nluc and the YFP-SOD1 were used as a positive and negative BRET control as described previously^9^. Nluc-block-YFP construction was generated upon introduction of the large rigid large portion of the TNF receptor-associated factor 2 (TRAF2, GeneID:7186) encoding sequence (233–501) flanked by Nluc and YFP sequence.

Gene identifiers used as reference to clone human genes were listed in Table 1.

**Table 1:** Human gene expression vectors.

Acceptor plasmid	Protein fusion name	Gene name	Gene ID
pEYFP-C1-p105	Y-p105	p105	4790
pEYFP-C1-p50	Y-p50	p50	4790
pEYFP-C1-RelA/p65	Y-RelA/p65	RelA/p65	5970
pEYFP-C1-RelA/p43	Y-RelAp43	RelA/p43	5970
pEYFP-C1-Nemo	Y-NEMO	NEMO (IKBKG)	8517
pEYFP-C1-TPL2	Y-TPL2	TPL2	1326
pEYFP-C1-STAT1	Y-STAT1	STAT1	6772
pEYFP-C1-STAT2	Y-STAT2	STAT2	6773
pEYFP-C1-STAT3	Y-STAT3	STAT3	6774
pEYFP-C1-JAK1	Y-JAK1	JAK1	3716
pEYFP-C1-IRF9	Y-IRF9	IRF9	10,379
pEYFP-C1-SOD1	Y-SOD1	SOD1	6647
pEYFP-C1-CAMKK2	Y-CAMKK2	CAMKK2	10,645
pEYFP-C1-FYN	Y-FYN	FYN	2534
pEYFP-C1-FKBP	Y-FKBP	FKBP1A	2280
pEYFP-N1-JAK1	JAK1-Y	JAK1	3716

Donor Plasmid	Protein fusion name	Gene name	Gene ID
pNluc-C1-p105	Nluc-p105	p105	4790
pNluc-C1-p50	Nluc-p50	p50	4790
pNluc-C1-TPL2	Nluc-TPL2	TPL2	1326
pNluc-C1-RelAp43	Nluc-RelAp43	RelAp43	5970
pNluc-C1-ABIN2	Nluc-ABIN2	ABIN2 (TNIP2)	79,155
pNluc-N1-SOD1	SOD1-Nluc	SOD1	6647
pNluc-N1-FRB	FRB-Nluc	FRB	2075
pNluc-C1-IFNAR2	Nluc-IFNAR2	IFNAR2	3455
pNluc-C1-STAT1	Nluc-STAT1	STAT1	6772
pNluc-C1-STAT2	Nluc-STAT2	STAT2	6773

### Cell culture, transfection, and treatment

HEK-293T cells (HEK-293T/17; ATCC CRL-11268) were maintained in Dulbecco’s modified Eagle’s medium (Gibco) supplemented with 10% fetal bovine serum (Gibco), 100 units/mL penicillin, and 0.1 mg/mL streptomycin at 37 °C in a 5% CO_2_ humidified atmosphere. One day prior to transfection, 4000 cells were seeded in 384-well flat (F) bottom μClear plates (Greiner, Bio One GmbH) coated with fibronectin (BD Biosciences). Transfection of HEK-293T cells was performed at 80% confluence using 25 ng of the total mixed plasmid(s) with 150 nL of Fugene 6 (Promega) reagent per well. For fixed and variable donor BRET saturation assays, HEK-293T were co-transfected with acceptor YFP- and donor Nluc-tagged genes of interest at 11 ratios ranging from 243:1 to 1:243 (Supplementary Fig. S2). After 48 h, protein expression was quantitatively assessed using fluorescence microscopy and bioluminescence readout. If indicated, cells were treated at 500 U/mL with recombinant human IFN-beta 1a (IFNβ) (PBL Assay Science, Cat 11415-1), or with 0.46 to 3000 nM of rapamycin (Seleckchem).

### Microscopy, net BRET, and normalization

Fluorescence cell imaging of live HEK-293T cells expressing YFP recombinant proteins seeded on 384-well flat (F) bottom μClear plates was performed with a 20 ×-magnifying lens, using an automated confocal microscope such as the Opera (PerkinElmer) or Operetta CLS (PerkinElmer). Nine images per well were acquired and processed with an in-house software named Image Mining^25^ to quantify the average fluorescence intensity per well. After the images had been taken with the automated confocal microscope, cells were kept in the CO_2_ incubator for 20 min prior to performing the bioluminescence acquisition. In order to avoid light absorption from the media, the HEK-293T cells culture media was next replaced by Dulbecco’s modified Eagle’s medium phenol red-free supplemented with Nano-Glo luciferase assay substrate containing furimazine, a cell-permeable substrate at 200-fold dilution (Promega, Madison, WI, USA). Direct bioluminescence from the donor and acceptor BRET channels were acquired sequentially using the Victor 3 V (Perkin Elmer), or the EnVision multilabel plate reader (Perkin Elmer) or the FluoSTAR (BMG Labtech), all harboring emission filters with the same band pass (BP) (λ_BP_ = 460/25 and λ_BP_ = 535/25 nm), with an acquisition time of 100 ms.

BRET value (absolute net BRET) were corrected considering the donor bleed-through to BRET channel for each sample using the equations below^26–28^. For each microplate, the bioluminescent (BL) signal was measured from cells expressing the donor molecule only (Nluc) using donor (λ_BP_ = 460/25 nm), and acceptor (λ_BP_ = 535/25 nm) filters in order to calculate the correction factor (cf). Next, the bleed-through signal emitted from the donor Nluc to the BL acceptor was corrected using the cf to calculate the absolute net BRET.

$${\text{cf}} = \frac{{{\text{BL }}\left( {\text{Acceptor filter}} \right){\text{ donor only}}}}{{{\text{BL }}\left( {\text{Donor filter}} \right){\text{ donor only }}}}$$

$${\text{absolute net BRET}} = \frac{{\left[ {{\text{BL }}\left( {\text{Acceptor filter}} \right) - {\text{cf}} \times {\text{BL }}\left( {\text{Donor filter}} \right)} \right]}}{{{\text{BL }}\left( {\text{Donor filter}} \right){ }}}$$

### Emission spectral scan

Emission spectral scans of the HEK-293T cells expressing recombinant Nluc fusions were performed in 384-well F bottom Clear plates using a SpectraMax M5 fluorescence microplate reader (Molecular Devices, CA), with Dulbecco’s modified Eagle’s medium without phenol red, supplemented with furimazine and the assay substrate Nano-Glo at 100-fold dilution (Promega). Emission spectra were recorded from 380 to 650 nm using an integration time of 500 ms with 5-nm step increments. All spectra were normalized to the luminescence value at the emission maximum (449 nm) of Nluc.

### Protein expression ratio and normalization

Expression levels of YFP tagged acceptor proteins were performed with an automated confocal Opera (Perkin Elmer) using an excitation (λ_ex_ = 488 nm) and a band-pass (λ_BP_ = 500–550 nm) emission filter. For quantification of the expression of the donor protein (Nluc), we used the sum of the bioluminescence signal obtained from the donor (λ_BP_ = 460/25 nm), and acceptor (λ_BP_ = 535/25 nm) channels. In order to normalize the relative molecular expression ratio of the donor and acceptor proteins, we expressed an Nluc-block-YFP fusion protein for each experimental microplate. Since this protein does not show any BRET (Fig. 1D), the signal from donor and acceptor channels were assumed to correspond to the A:D molecular ratio of 1:1. As a result, using the Nluc-block-YFP fluorescent and BL signal, we were able to normalize the relative molecular ratio of acceptor and donor (YFP/Nluc) within each sample. As represented in the BRET saturation assays, the A:D expression ratios were converted in log value.

### Graphical representation and statistical analysis

Each experiment was performed at least in biological triplicate and technical duplicate. For each experiment, the values from each well were plotted independently for optimal fitting. If required for figure readability, identical data points were summarized into one average value. Figures, curves, and statistical analysis were performed in Prism (GraphPad). The curves were fitted according to a nonlinear regression equation assuming a single binding site. Non-interacting pairs were used to define a 3σ threshold (3σ = Negative controls _average_ + 3 × Negative controls_standard deviation_). Multiple comparisons of data were performed by ANOVA.

## Supplementary Information

Supplementary Legends.

Supplementary Figures.

## Data Availability

The data generated during this study are available from the corresponding author upon reasonable request.
