# Supplementary material for: Optimization of BRET saturation assays for robust and sensitive cytosolic protein–protein interaction studies
Source: Sci Rep. 2022 Jun 15;12:9987. doi: 10.1038/s41598-022-12851-9 (PMC9200754; doi:10.1038/s41598-022-12851-9)
Supplement: Supplementary file 2 — Supplementary Figures. [file 41598_2022_12851_MOESM2_ESM.pdf]

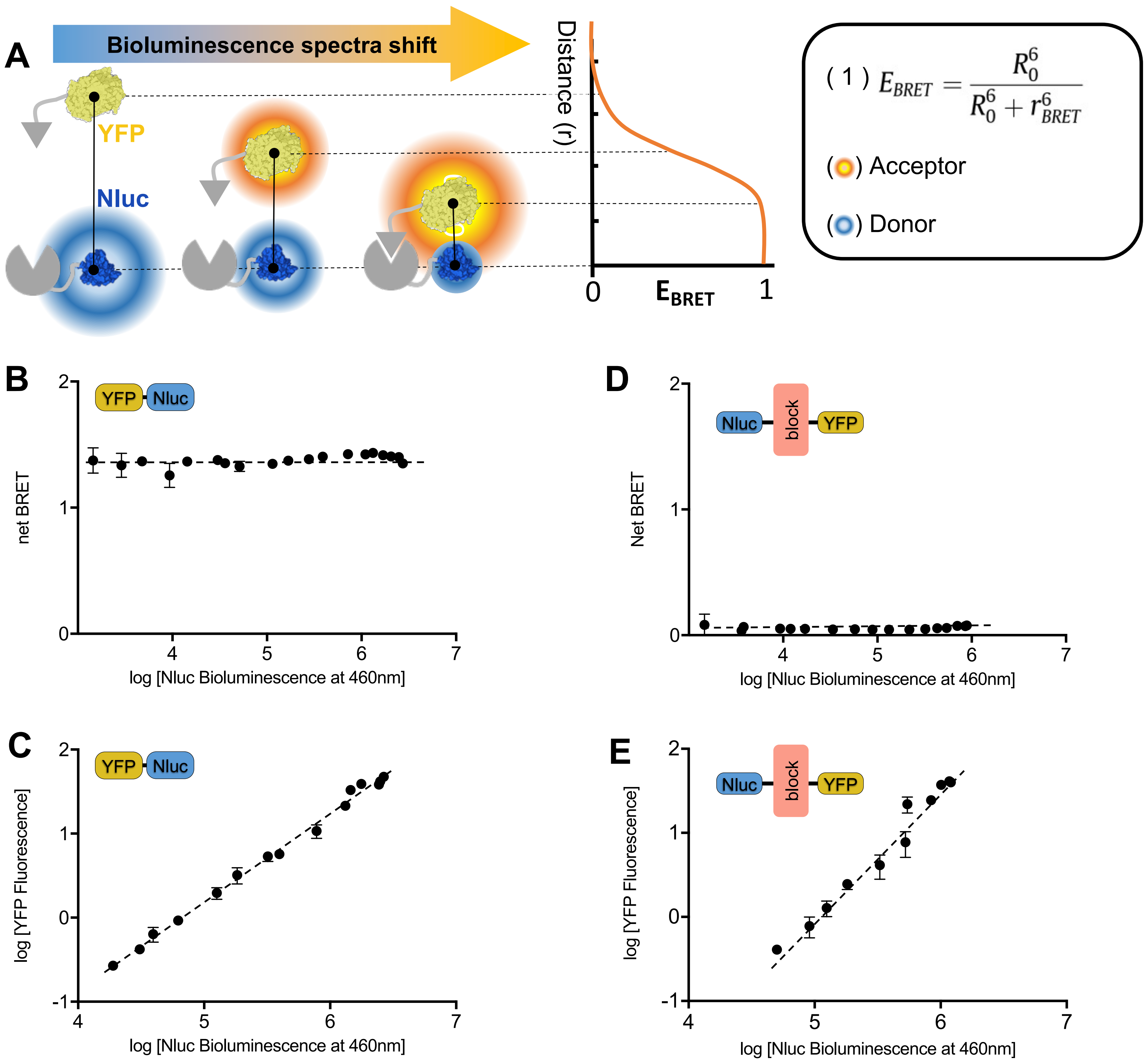

Supplementary Figure 1. Control experiment to validate the linearity between bioluminescence and fluorescence measurement as well as net BRET stability.

A. Diagram of bioluminescence resonance energy transfer. Representation of the dependence of the bioluminescence energy transfer efficiency (E_{BRET}) with the distance (r) between the donor (Nluc) and acceptor molecule (YFP) leading to bioluminescence spectra shift. The Förster equation (1) specifies that BRET efficiency (E_{BRET}) is proportional to the sixth power of the ratio between the actual donor/acceptor distance (r) and the Förster radius (R_0) corresponding to the distance leading to 50% of the energy transferred between BRET pairs. **B-E.** Upon fluorescence excitation at 488nm of YFP-Nluc and Nluc-stop-YFP probes, YFP emission is measured at 527 nm (Operetta). In the presence of furimazine, two bioluminescence signals are measured using Victor3 from YFP-Nluc, the Nluc (blue: 460nm) and BRET (yellow: 510nm), while only one signal is measured from the Nluc-stop-YFP. **B-C, D-E.** HEK-293 cells were transfected for 48h with various ratios of YFP-Nluc or Nluc-stop-YFP encoding vector and mock plasmid at a constant total DNA of 25 ng. Stability of the absolute net BRET (**B, D**) and linear relationship between Nluc bioluminescence and YFP fluorescence (**C, E**) of the YFP-Nluc and Nluc-stop-YFP at various expression levels.

A

Fix D & variable A	1	2	3	4	5	6	7	8	9	10	11
**Donor (ng)**	0.2	0.2	0.2	0.2	0.2	0.2	0.2	0.2	0.2	0.2	0.2
**Acceptor (ng)**	0.0008	0.0025	0.007	0.02	0.07	0.2	0.6	1.8	5.4	16.2	48.6
**Mock (ng)**	49.7992	49.7975	49.793	49.78	49.73	49.6	49.2	48	44.4	33.6	1.2
**Ratio A:D**	**0.004**	**0.012**	**0.037**	**0.111**	**0.333**	**1.000**	**3.000**	**9.000**	**27.000**	**81.000**	**243.000**

B

Variable D & A	1	2	3	4	5	6	7	8	9	10	11
**Donor (ng)**	24.9	24.7	24.1	22.5	18.7	12.5	6.3	2.5	0.9	0.3	0.1
**Acceptor (ng)**	0.1	0.3	0.9	2.5	6.3	12.5	18.8	22.5	24.19	24.7	24.9
**Ratio A:D**	**0.004**	**0.012**	**0.037**	**0.111**	**0.333**	**1.000**	**3.000**	**9.000**	**27.000**	**81.000**	**243.000**

C

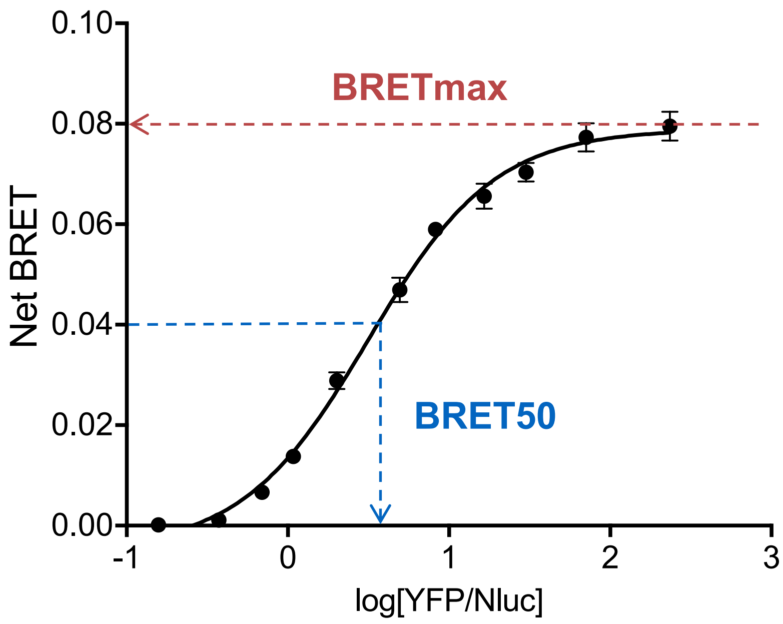

Supplementary Figure 2. Fixed or variable donor BRET saturation method.
HEK-293 cells were transfected with a mix of acceptor expressing plasmids diluted with 11 conditions. **A.** For conditions using a constant amount of donor we used 0.2ng of donor plasmid and variable levels of acceptor (0.0008 to 48.6ng) and completed each condition with a Mock plasmid to obtain a fixed amount of 50 ng of DNA to be transfected per well of 384 well plates. **B.** For conditions using variable amounts of donor and acceptor, we used variable levels of acceptor and donor (0.1 to 24.9 ng) for a total amount of 25 ng of DNA to be transfected per well of 384 well plates. After 48h, YFP and Nluc expression ratio and net BRET were plotted and fitted using a non-linear regression curve. **C.** BRET saturation curve exploitation for BRETmax and BRET50 calculation. BRETmax is the maximal net BRET signal observed at high A:D expression ratios. BRET50 is the A:D expression ratio allowing the net BRET to reach 50% of BRETmax.

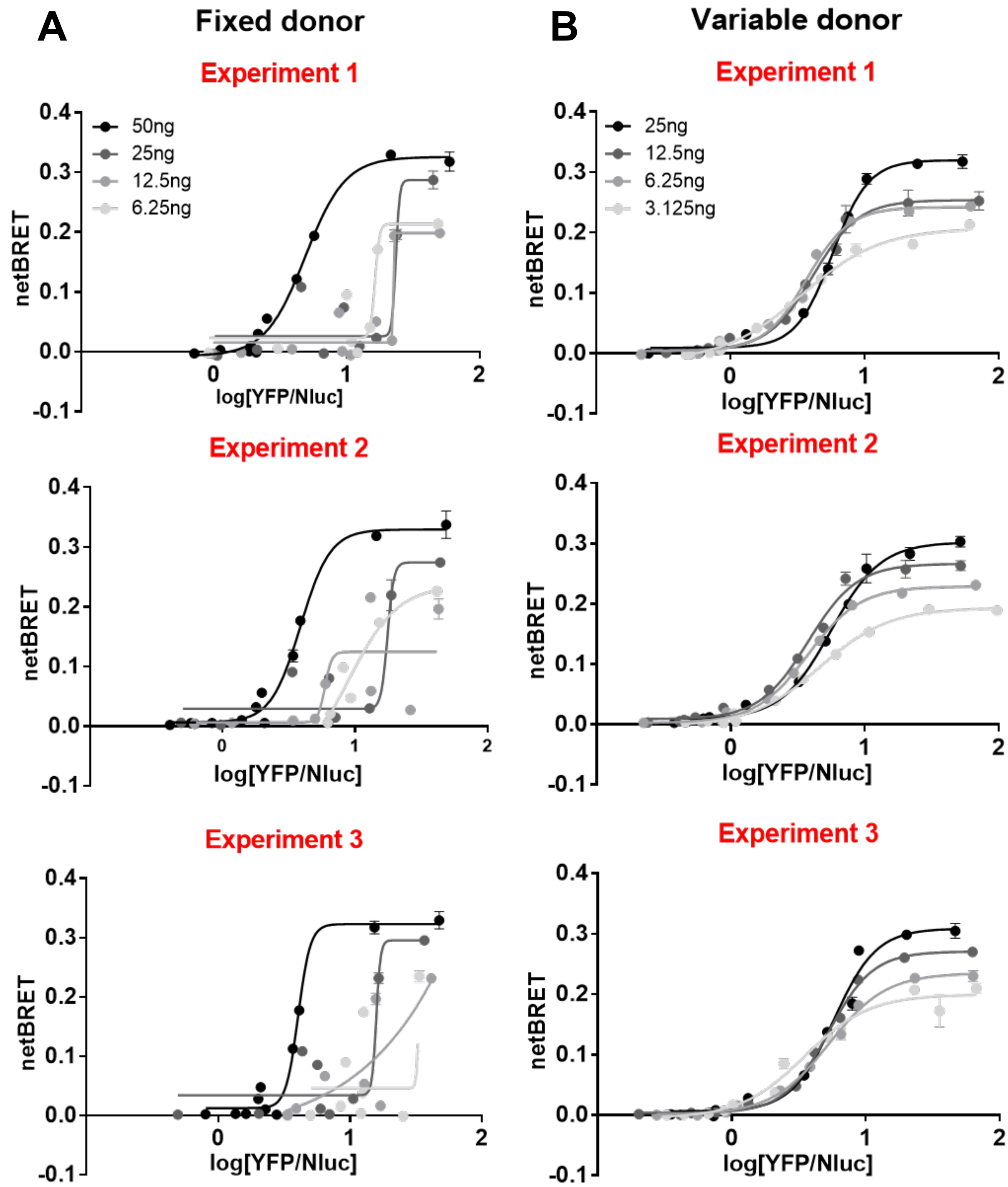

Supplementary Figure 3. Reliability of variable over fixed donor BRET saturation assays

A-B. HEK-293 cells were co-transfected for 48 h with the indicated total amount of plasmids encoding 11 ratios (243:1 to 1:243) using fixed (**A**) and variable donor (**B**) expression of Nluc-p50 with YFP-tagged p50. Bioluminescence (blue wavelength) and fluorescence (yellow wavelength) were measured with a plate reader or a confocal microscope, respectively. It should be noted that the BRET saturation assay based on the transfection of a fixed donor was prone to failure when transfecting lower amounts of total plasmids. In contrast, the variable donor method reliably reached a proper signal saturation in all conditions. Each panel represent individual biological replicates, each constituted of 3 technical replicates.

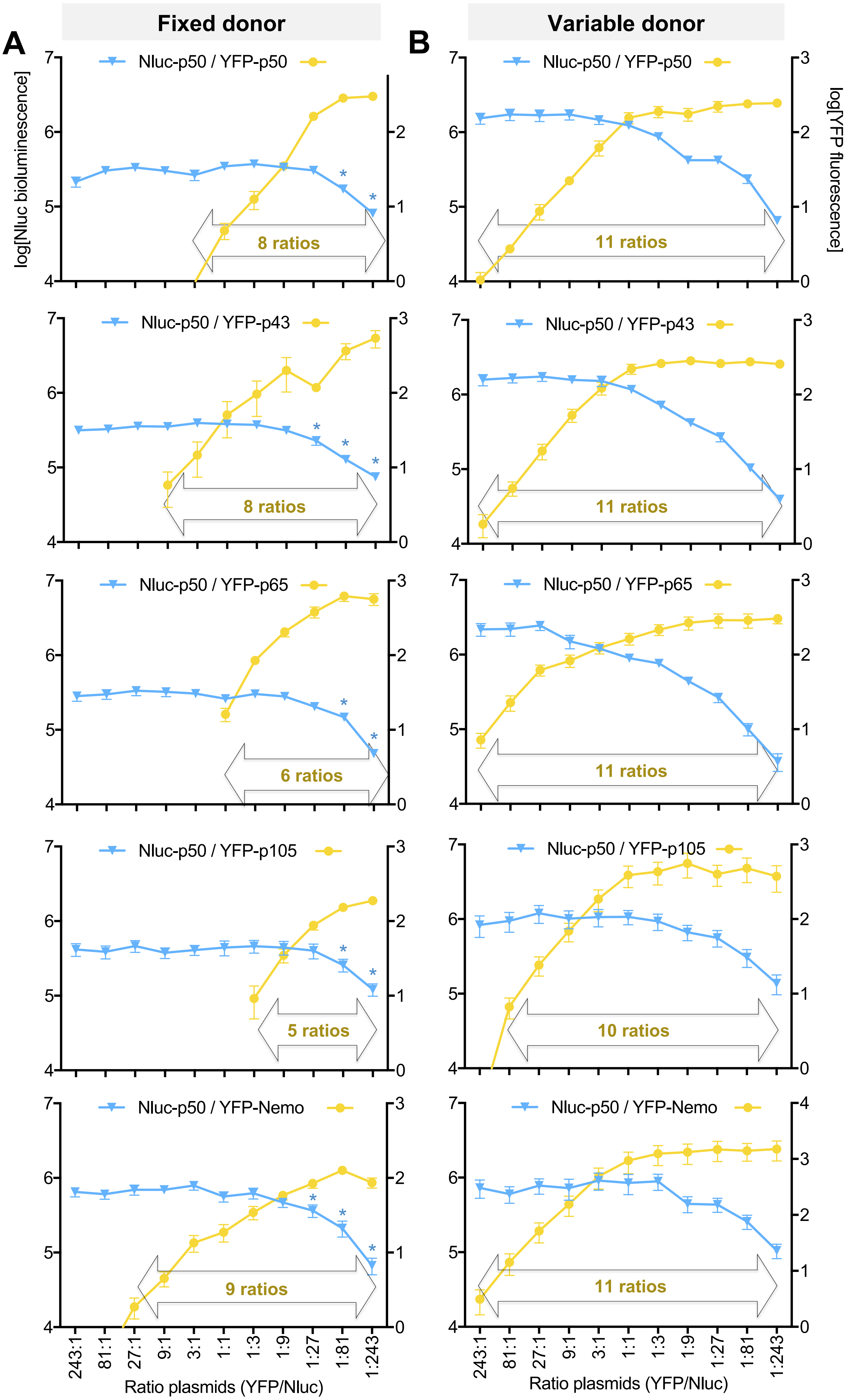

Supplementary Figure 4. Protein expression quantification upon fixed or variable donor BRET saturation.
A-B. HEK-293 cells were co-transfected for 48 h with plasmids encoding 11 ratios (243:1 to 1:243) using fixed (**A**) and variable donor (**B**) expression of Nluc-p50 with YFP-tagged p50, p43, p65, p105, or NEMO (see Fig. 1H-I). Bioluminescence (blue) and fluorescence (yellow) were measured using a plate reader or a confocal microscope, respectively. It should be noted that for the fixed donor BRET saturation protocol (**B**) the Nluc bioluminescent signal significantly decreased for high A:D plasmid ratio. *P < 0.05.

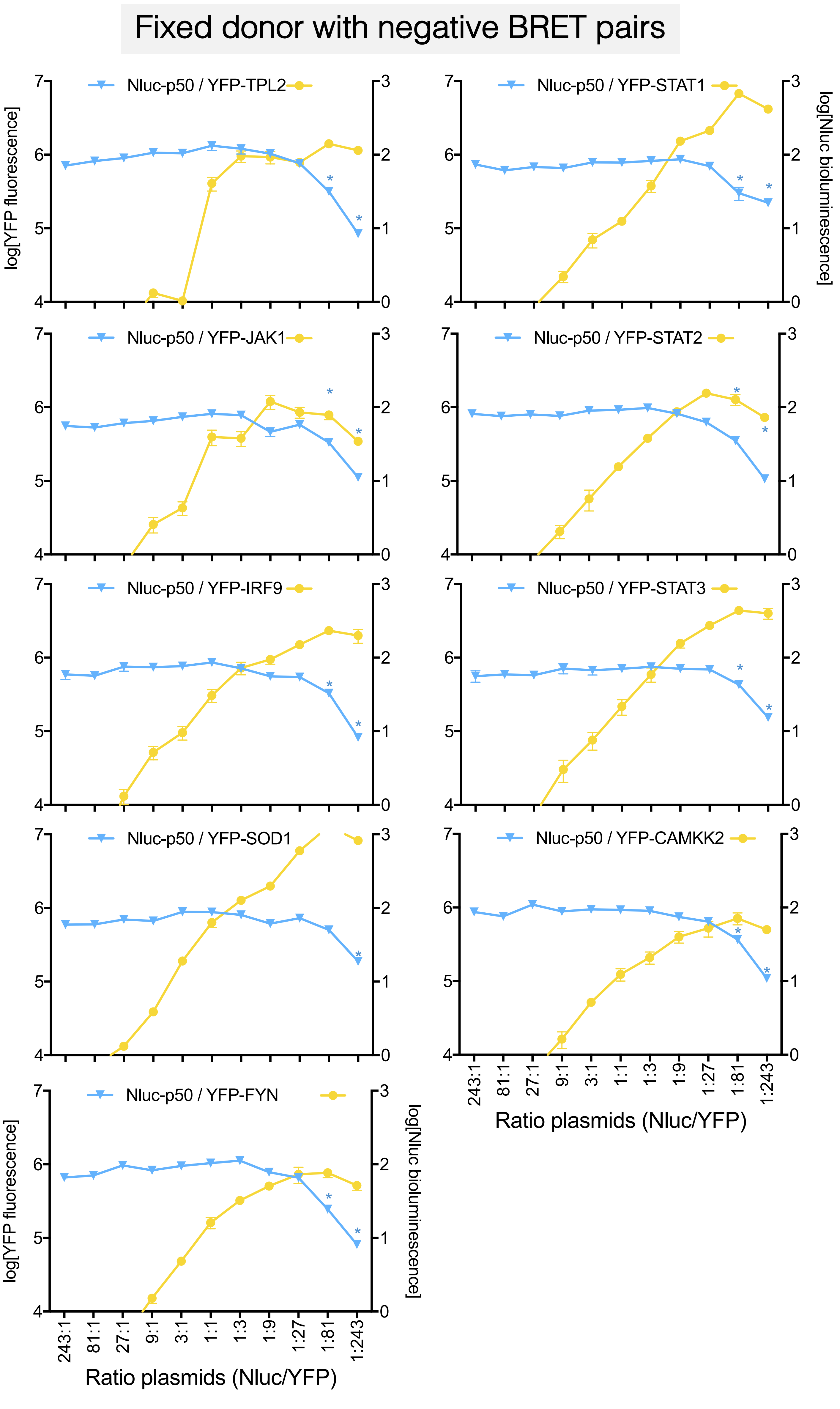

Supplementary Figure 5. Protein expression quantification of non-interacting pairs upon fixed donor BRET saturation.
HEK-293 cells were co-transfected for 48 h with plasmids encoding 11 ratios (243:1 to 1:243) using fixed donor expression of Nluc-p50 with YFP-tagged TPL2, STAT1, STAT2, STAT3, JAK1, IRF9, SOD1, CAMKK2 and FYN (see Fig. 1H). Bioluminescence (blue wavelength) and fluorescence (yellow wavelength) were measured using a plate reader or a confocal microscope, respectively. It should be noted that the Nluc bioluminescent signal significantly decreased for a high A:D plasmid ratio. * $p < 0.05$.

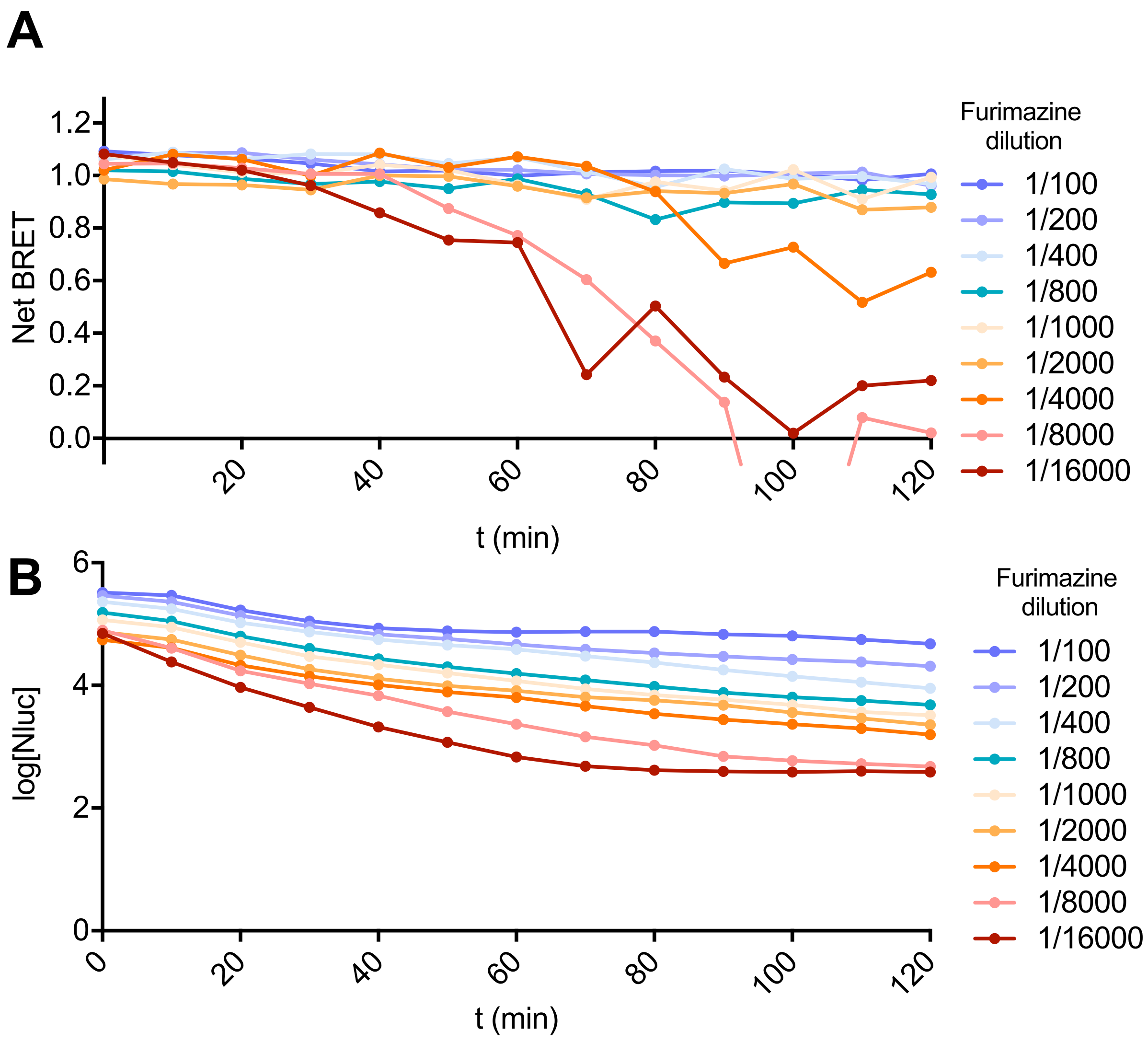

Supplementary Figure 6. Control experiment to validate the measurement of net BRET over time and the effect of furimazine concentration.
HEK-293 cells were transfected for 48 h with 25 ng of YFP-Nluc recombinant protein. **A.** Absolute net BRET signal and **B.** bioluminescence signal of Nluc were monitored over 2 h after an initial treatment with various dilutions of furimazine (1/25-1/16000).
